# Cardioprotective Effect of *Ulmus wallichiana* Planchon in β-Adrenergic Agonist Induced Cardiac Hypertrophy

**DOI:** 10.3389/fphar.2016.00510

**Published:** 2016-12-21

**Authors:** Anees A. Syed, Shibani Lahiri, Divya Mohan, Guru R. Valicherla, Anand P. Gupta, Sudhir Kumar, Rakesh Maurya, Himanshu K. Bora, Kashif Hanif, Jiaur R. Gayen

**Affiliations:** ^1^Division of Pharmacokinetics and Metabolism, Council of Scientific and Industrial Research–Central Drug Research InstituteLucknow, India; ^2^Division of Pharmacology, Council of Scientific and Industrial Research–Central Drug Research InstituteLucknow, India; ^3^Academy of Scientific and Innovative ResearchNew Delhi, India; ^4^Division of Medicinal and Process Chemistry, Council of Scientific and Industrial Research–Central Drug Research InstituteLucknow, India; ^5^Division of Laboratory Animals, Council of Scientific and Industrial Research–Central Drug Research InstituteLucknow, India

**Keywords:** *Ulmus wallichiana*, ethnopharmacology, cardiac hypertrophy, cardiovascular disease, isoprenaline, blood pressure, heart rate

## Abstract

*Ulmus wallichiana* Planchon (Family: Ulmaceae), a traditional medicinal plant, was used in fracture healing in the folk tradition of Uttarakhand, Himalaya, India. The present study investigated the cardioprotective effect of ethanolic extract (EE) and butanolic fraction (BF) of *U. wallichiana* in isoprenaline (ISO) induced cardiac hypertrophy in Wistar rats. Cardiac hypertrophy was induced by ISO (5 mg/kg/day, subcutaneously) in rats. Treatment was performed by oral administration of EE and BF of *U. wallichiana* (500 and 50 mg/kg/day). The blood pressure (BP) and heart rate (HR) were measured by non-invasive blood pressure measurement technique. Plasma renin, Ang II, NO, and cGMP level were estimated using an ELISA kit. Angiotensin converting enzyme activity was estimated. BP and HR were significantly increased in ISO group (130.33 ± 1.67 mmHg vs. 111.78 ± 1.62 mmHg, *p* < 0.001 and 450.51 ± 4.90 beats/min vs. 347.82 ± 6.91 beats/min, respectively, *p* < 0.001). The BP and HR were significantly reduced (EE: 117.53 ± 2.27 mmHg vs. 130.33 ± 1.67 mmHg, *p* < 0.001, BF: 119.74 ± 3.32 mmHg vs. 130.33 ± 1.67 mmHg, *p* < 0.001); HR: (EE: 390.22 ± 8.24 beats/min vs. 450.51 ± 4.90 beats/min, *p* < 0.001, BF: 345.38 ± 6.79 beats/min vs. 450.51 ± 4.90 beats/min, *p* < 0.001) after the treatment of EE and BF of *U. wallichiana*, respectively. Plasma renin, Ang II, ACE activity was decreased and NO, cGMP level were increased. The EE and BF of *U. wallichiana* down regulated the expression of ANP, BNP, TNF-α, IL-6, MMP9, β1-AR, TGFβ1 and up regulated NOS3, ACE2 and Mas expression level, respectively. Thus, this study demonstrated that *U. wallichiana* has cardioprotective effect against ISO induced cardiac hypertrophy.

## Introduction

Cardiac hypertrophy is the abnormal growth, or thickening, of the heart muscle, often due to chronic hypertension that leads to HF. While in left ventricular hypertrophy, the myocardial oxygen consumption is increased which results in decreased coronary blood flow reserve which results in angina pectoris, myocardial infarction, and sudden death (Levy et al., 1990). It is estimated that nearly 23 million people are affected by HF worldwide (McMurray et al., 1998). In India, the prevalence of HF is around 4.6 million with an annual occurrence of nearly 1.8 million (Huffman and Prabhakaran, 2010). Cardiac hypertrophy is a physiological adaptation which causes increased workload on the myocardium. Hypertrophic growth is due to many types of heart disease, including ischaemic heart disease, hypertension, heart failure, cardiac hypertrophy, and valvular disease (Frey et al., 2004).

Many bioactive compounds have been discovered from plants which have flavonoids, natural phenolic compounds, and phytoestrogens are strong antioxidant (Horáková, 2011; Yan et al., 2013). Fruits and vegetables which are enriched with higher content of flavonoid prevents the development of cardiovascular disease (Horáková, 2011). Cardiac hypertrophy is inhibited by different antioxidants caused by ROS (Tsujimoto et al., 2005; Sheng, 2007). Quercetin (a polyphenolic flavonoid); and its analogs and its major metabolite modulate NO/cGMP-dependent vasorelaxation and regulate BP (Suri et al., 2010). Flavonoids such as Quercetin (Jalili et al., 2006; Han et al., 2009; Yan et al., 2013); Pentamethylquercetin (He et al., 2012); Hesperetin (Deng et al., 2013); Curcumin (Li et al., 2008); Epigallocatechin-3 gallate (EGCG) (Hao et al., 2007); Myricetin (Tiwari et al., 2009) possess cardioprotective effect.

*Ulmus wallichiana* Planchon (Family: Ulmaceae), a traditional Indian medicinal plant used as astringent, demulcent, emollient, expectorant and diuretic (Khare, 2008). It contains quercetin analog flavonoids (2S,3S)-(+)-3′,4′,5,7-tetrahydroxydihydroflavonol-6-C-β-D-glucopyranoside **(K058)**; 6-Glucopyranosyl-3,3′,4′,5,7-pentahydroxyflavone **(K012)**; 6-Glucopyranosyl-4′,5,7-trihydroxyflavanone **(K068)**, and (2S,3S)-(+)-4′,5,7-trihydroxydihydroflavonol-6-C-β-D-glucopyranoside **(K100)** of known chemical structures (Supplementary Figure 1) and composition (Supplementary Table 1) (Rawat et al., 2009; Sharan et al., 2010). The characterization of compounds present in EE and BF of *U. wallichiana* namely K012, K058, K068, and K100 was reported by Maurya et al. (2014). These flavonoids are potent for anabolic effect on osteoporotic bone (Sharan et al., 2011), increases bone mass density, bone strength, and bone formation rate which enhances osteoblast differentiation and achieves peak bone in growing rats and has osteoprotective effect on ovariectomized rats (Sharan et al., 2010). It also lowers blood glucose level in diabetic rats (Rawat et al., 2011). The EE and BF of *U. wallichiana* contains quercetin analog flavonoids (Sharan et al., 2010).

*Ulmus wallichiana* is used traditionally as diuretic (Khare, 2008). A related species *U. rhynchophylla*, native to China, known as Gou Teng in Chinese medicine, is used for eclampsia, headache, dizziness, convulsions, high fever, and hypertension (WHO). *U. macrocarpa* has antihypertensive and vasorelaxant activity (Oh et al., 2008). Therefore due to diuretic property of *U. wallichiana* it is used in hypertension as it contains quercetin (a polyphenolic flavonoid) and its major metabolites modulate NO/cGMP dependent vasorelaxation and regulates BP (Suri et al., 2010). Due to prolong increase in BP (hypertension) there is direct haemodynamic stress, leading to hypertrophy (Mehta et al., 2014). Therefore, we have explored the effect of EE and BF of *U. wallichiana* in cardiac hypertrophy.

The effective dose of EE ranges from 500 to 750 mg/kg and that of BF ranges from 25 to100 mg/kg as reported in patent by Maurya et al. (2014) (co-author of this article). We have selected the above dose of EE (500 mg/kg) as it showed optimal decrease in BP. As this dose is high enough to be translated from rat to human, BF was isolated and used with effective dose of 50 mg/kg. BF enriched with high content of quercetin analog flavonoids compared to EE (Sharan et al., 2010). Therefore the effective dose of EE is 500 mg/kg and that of BF is 50 mg/kg (Maurya et al., 2014).

The anti-hypertrophic activity of *U. wallichiana* was remained unexplored in cardiac hypertrophy model. In our lab, we have explored the antihypertensive activity of EE and BF of *U. wallichiana* (Syed et al., 2016). Therefore, in the present work, we have investigated the anti-hypertrophic activity of EE and BF of *U. wallichiana* in ISO induced cardiac hypertrophy.

## Materials and Methods

### Chemicals and Reagents

Isoprenaline hydrochloride, Olive oil, Gum Acacia, *N*-[3-(2-Furyl) acryloyl]-L-phenylalanyl-glycyl-glycine (FAPGG), 2′,7′-Dichlorofluorescein diacetate and TRIzol reagent were procured from Sigma–Aldrich Chemical Co., St. Louis, MO, USA. Sodium Chloride was procured from Merck Specialities Private Limited, Mumbai, India. Tris (Hydroxymethyl) Aminomethane was procured from SRL Pvt. Ltd., Mumbai, India. High capacity RNA to cDNA Reverse Transcriptase kit was procured from Applied Biosystems, USA. 2X SYBR green Premix Ex Taq (Takara Bio, Japan), List of rat primers for Real-Time (RT) qPCR (Eurofins Scientific, Luxembourg) as shown in **Table 1**, Renin ELISA Kit (Shanghai, China), Human/Mouse/Rat Angiotensin II Enzyme Immunoassay Kit (RayBiotech, Inc.), Nitrate/Nitrite Colorimetric Assay Kit (Cayman Chemical, Ann Arbor, MI, USA), cGMP Assay Kit (Cayman Chemical, Ann Arbor, MI, USA), Renin Inhibitor Screening Assay Kit (Cayman Chemical, Ann Arbor, MI, USA) were purchased.

**Table 1:** Primers of cardiac hypertrophy related genes for RT qPCR.

Gene	Forward primer	Reverse primer
rANP	GAGGAGAAGATGCCGGTAG	CTAGAGAGGGAGCTAAGTG
rBNP	TGATTCTGCTCCTGCTTTTC	GTGGATTGTTCTGGAGACTG
rTNF-α	CCCAGACCCTCACACTCAGAT	TTGTCCCTTGAAGAGAACCTG
rIL-6	CCCTTCAGGAACAGCTATGAA	ACAACATCAGTCCCAAGAAGG
rNOS3	AAAGGAAGTGCAGCAAAAGG	CCCATGAGTGAGGCAGAGAT
rTGFβ1	CCTGGAAAGGGCTCAACAC	CAGTTCTTCTCTGTGGAGCTGA
rACE2	TCAAGGGAAAAGAACCAGACA	GGTTTCAAATCACTCACCCATAC
rAdrb1	AGAGCAGAAGGCGCTCAAG	AGCCAGCAGAGCGTGAAC
rMas1	ACGTCCCCAGACCAGTCAT	TGAGGAGTTCTTGTGCTGGA
rMMP9	CCTCTGCATGAAGACGACATAA	GGTCAGGTTTAGAGCCACGA
rGAPDH	TGGGAAGCTGGTCATCAAC	GCATCACCCCATTTGATGTT

### Plant Material

Stem bark of *U. wallichiana* was collected and authenticated by Arya et al. (2013) of CSIR–Central Drug Research Institute (CDRI), Lucknow, India and extraction procedure was reported (Sharan et al., 2010; Arya et al., 2013; Maurya et al., 2014). EE and BF from stem bark of *U. wallichiana* were obtained from Medicinal and Process Chemistry division of CSIR–CDRI, Lucknow, India. The fingerprinting of EE and BF of *U. wallichiana* was done by us using LC-MS/MS – Mass spectrometer QTrap 5500-AB (Applied Biosystems/MDS Sciex, Canada), using an electrospray ionization source in negative ion mode (ESI) and published (Syed et al., 2016).

### Animals

The experiments were carried out with male normotensive wistar rats (180–220 g) procured from the Laboratory Animal Division of CSIR–CDRI, Lucknow, India. Experiments were performed according to protocols approved by Institutional Animal Ethics Committee [(IAEC) no. IAEC/2012/92N] of CSIR–CDRI and Committee for the Purpose of Control and Supervision of Experiments on Animals (CPCSEA), India. Rats were maintained under standard housing conditions (room temperature 25 ± 2°C and humidity 60 ± 5%) with a 12 h light and dark cycle. Food and water were available *ad libitum*.

### Study Protocols

Male wistar rats were injected subcutaneously (s.c.) with ISO 5 mg/kg/day for 10 days (Yeh et al., 2010) with or without 500 mg/kg/day of EE and 50 mg/kg/day of BF of *U. wallichiana*. The rats were randomly divided into four groups and each group contains six animals each.

(i) Control group: olive oil (vehicle) was given s.c. for 10 days.
(ii) ISO group: Isoprenaline, 5 mg/kg/day was administered subcutaneously for 10 days, to induce cardiac hypertrophy.
(iii) ISO + EE group: EE was administered orally for 14 days at a dose of 500 mg/kg/day of *U. wallichiana* after development of cardiac hypertrophy.
(iv) ISO + BF group: Oral administration of BF for 14 days at a dose of 50 mg/kg/day of *U. wallichiana* after development of cardiac hypertrophy.

### Blood Pressure Measurement

#### NIBP Measurement

To measure changes in arterial blood pressure in conscious rat, NIBP technique was used (Columbus instrument, Model NIBP-8). Conscious rats were acclimatized in the restrainer for 15 min three times a day for a period of 1-week before the starting of the testing schedule. The trained animals were placed in a restrainer and tails of rats were exposed to hot air blower. The rats were allowed to acclimatize inside the cage for 15 min before starting the actual blood pressure measurement. Occlusion and sensor cuffs were placed around the base of the tail and the cuffs were inflated and deflated several times to measure tail arterial blood pressure. A minimum of 8–10 cycles of inflation and deflation were made and the measurement of tail arterial blood pressure was recorded and the data was averaged. The data were recorded and analyzed with software.

#### IBP Measurement

Arterial blood pressure was measured by invasive method in the urethane (1.2 g/kg) anesthetized rats. The tracheotomy was performed. The catheter was inserted into the carotid artery to monitor the alteration in the arterial blood pressure. The other end of the catheter was connected to the computerized Data Acquisition System (DAS), AD-Instruments with compatible software.

### Determination of Heart Weight to Body Weight Ratio, Heart Weight to Tail Length Ratio, and Heart Weight to Tibia Length Ratio

After the measurement of IBP the rats were sacrificed and heart weight, tail length, and tibia length were measured. The heart weight index was calculated by dividing the heart weight by the body weight (Yeh et al., 2010), the heart tail index was calculated by dividing the heart weight by tail length (Chowdhury et al., 2013), and the heart tibia index was calculated by dividing the heart weight by tibia length (Oh et al., 2015).

### Renin Inhibition Assay

The renin inhibition assay was performed by Renin Inhibitor Screening Assay Kit (catalog number: 10006270; Chemical, Ann Arbor, MI, USA) according to the manufacturer’s instructions. The renin inhibition was expressed as percentage inhibition or percent initial activity as a function of the inhibitor concentration to determine the IC_50_ value.

### Estimation of Plasma Renin Level in Plasma

The plasma renin assay was measured by rat Renin (Renin) ELISA kit (catalog number: E02R0022; BlueGene Biotech, Shanghai, China) according to the manufacturer’s instructions. To determine renin level, the blood was collected from the retro–orbital plexus of rat in EDTA containing tubes. Plasma was harvested by using centrifugation at 1000 × *g* for 15 min at 4°C. Plasma (100 μL) was used for estimation of renin concentration.

### Angiotensin Converting Enzyme (ACE) Assay

Angiotensin converting enzyme activity was determined by a spectrophotometric mono-reagent assay. The reaction mixture containing serum (10 μL) and the substrate solution (200 μL) were kept at 37°C in Elisa plate reader (BIOTEK, USA) and reaction was monitored at 340 nm for 30 min. Serum ACE activity was expressed as unit/L (Hanif et al., 2009).

### Determination of Angiotensin II (Ang II) Level in Plasma

The plasma AG II level was estimated by Human/mouse/Rat Ang II Enzyme Immunoassay Kit (catalog#: EIA-ANGII, EIAM-ANGII, EIAR-ANGII; RayBiotech, Inc.) according to the manufacturer’s instructions.

### Estimation of Nitrate/Nitrite (NO) Level in Plasma

The plasma NO was estimated by Nitrate/Nitrite Colorimetric Assay Kit (catalog number: 780001; Cayman Chemical, Ann Arbor, MI, USA) according to the manufacturer’s instructions. To determine nitrate/nitrite level in plasma, 40 μL of plasma was taken and diluted with 40 μL of Assay Buffer to adjust the volume to 80 μL of sample and used for NO determination.

### Estimation of cGMP Level in Plasma

The cGMP concentrations were estimated using an enzyme immunoassay (EIA) kit (catalog number: 581021; Cayman Chemical, Ann Arbor, MI, USA), as per the manufacturer’s instructions. To determine the cGMP levels in plasma, 2 mL of ice-cold ethanol was added to 500 μL of plasma, and the solution was centrifuged at 1500 × *g* for 10 min. The supernatant was evaporated by vacuum centrifugation (Turbo Vap LV, Caliper, USA) and used for the cGMP estimation.

### Determination of Reactive Oxygen Species (ROS)

Reactive oxygen species was estimated in heart tissue. Heart tissue was homogenized in ice-cold 40 mMtris buffer (pH 7.4) and samples were centrifuged at 100,000 × *g* at 4°C in an Eppendorf centrifuge (5819 R Germany) for 15 min. The resulting heart homogenate was incubated with DCFDA (5 μM) for 30 min at 37°C. Formation of fluorescent product DCF was monitored by fluorometer at an excitation wavelength of 488 nm and emission wavelength of 530 nm.

### Histopathological Examination

After measurement of hemodynamic parameters of rats, their hearts were quickly and filled with 4% paraformaldehyde solution, and kept in it for 24 h. The heart samples were embedded in paraffin blocks. Samples were sliced of 5 μm thickness and stained with hematoxylin and eosin (H&E) for assessing myocardial fibrosis. Myocardial fibrosis and relative cell surface area were evaluated in each of the heart tissue sections using a morphometric procedure using ImageJ software.

### RNA Extraction and Relative Gene Expression Analysis by Real-Time PCR

#### Total RNA Extraction

Total RNA was extracted from 0.1 to 0.2 g of rat heart tissues by using TRIzol reagent as per manufacturer’s protocol. The quality and quantity of the isolated RNA were assessed using the 260/280 nm absorbance ratio (1.8–2.0 indicates a highly pure sample) using NanoDrop 2000c spectrophotometer (USA). Total RNA was stored at -80°C until used.

#### Synthesis of cDNA and RT qPCR Quantification

RNA was transcribed into cDNA in a 20 μL reaction using a High Capacity RNA to cDNA Reverse Transcriptase kit, analyzed, and amplified. 2x master mixes were prepared using 2 μl 10x Reverse Transcription buffers, 0.8 μL 25x dinucleotide triphosphate (dNTP) mix (100 mM), 2 μL 10x Reverse Transcription random primers, and 1 μL MultiScribe Reverse Transcriptase. For a 20 μL reaction mixture under the following conditions: 5.8 μL master mix; 2 μL of 2 μg RNA sample; 12.2 μL nuclease free water. Following was the program for reverse transcriptase PCR using SureCycler 8800 (Agilent Technologies, USA): 25°C for 10 min; 37°C for 120 min; 85°C for 5 min; hold at 4°C. The product obtained was diluted 4× for real-time PCR. Samples were seeded in triplicate in 96-well reaction plates (LightCycler 480). RT qPCR was performed on Light Cycler 480 II (Roche Diagnostics) with SYBR Green fluorescent label. Samples (10 μL final volume) contained the following: 2x SYBR Premix Ex Taq 1 μL of each primer; 3 μL of cDNA sample, and 5 μL of nuclease free water. Cycle threshold (Ct) values were used to calculate the amount of amplified PCR product relative to glyceraldehyde-3-phosphate dehydrogenase (GAPDH) as control (reference gene).

### Statistical Analysis

Statistical analysis was performed with Prism software version 5.01 (Graph Pad Software, San Diego, CA, USA) and Curve Expert Professional version 2.0.3. Results are expressed as mean ± SEM. Statistical significance was evaluated by one-way analysis of variance (ANOVA) followed by Newman–Keuls Multiple comparison test and *t*-test. A value of *p* < 0.05 was considered to be statistically significant.

## Results

### *U. wallichiana* Attenuated Heart Weight to Body Weight Ratio, Heart Weight to Tail Length Ratio, and Heart Weight to Tibia Length Ratio

**FIGURE 1:**
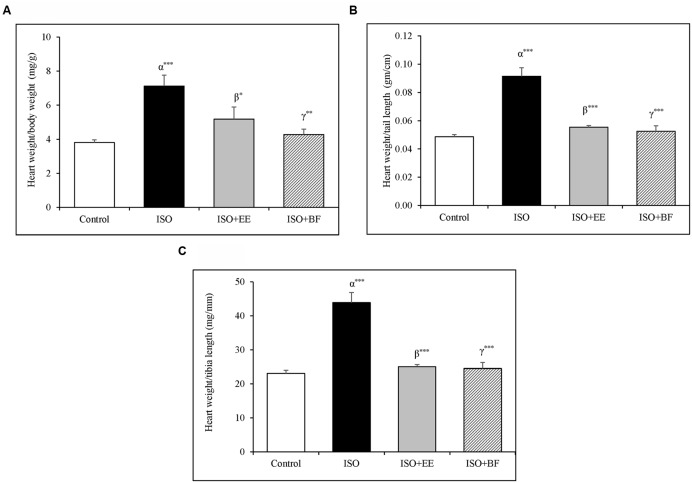
**Effect of *Ulmus wallichiana* on (A)** Heart weight to body weight ratio, **(B)** Heart weight to tail length ratio, and **(C)** Heart to tibia length ratio in ISO induced cardiac hypertrophy in wistar rats. Data were analyzed by using one-way ANOVA followed by Newman–Keuls Multiple comparison test. Data are shown as Mean ± SEM; α, control vs. ISO; β, ISO vs. EE; γ, ISO vs. BF; ^∗^*P* < 0.05, ^∗∗^*P* < 0.01, ^∗∗∗^*P* < 0.001.

Heart to body weight, heart weight to tail length, and heart weight to tibia length ratio were significantly increased in the ISO groupas compared to control group. The treatment of extract and fraction of *U. wallichiana* significantly decreased heart to body weight, heart weight to tail length, and heart weight to tibia length ratio in ISO+EE and ISO+BF group compared to disease control (**Figures 1A–C**), respectively. The images of whole heart have been provided in the Supplementary Figure 3.

### Hemodynamics

**FIGURE 2:**
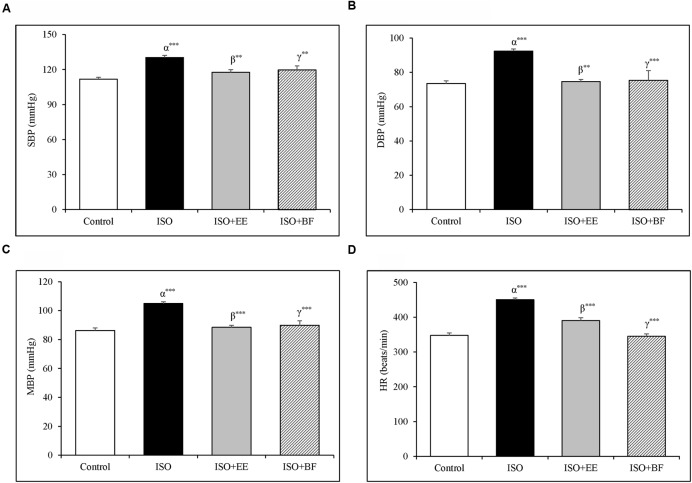
**Effect of *U. wallichiana* on BP (A)** SBP, **(B)** DBP, **(C)** MAP, and **(D)** HR by NIBP measurement technique in ISO induced cardiac hypertrophy in wistar rats. Data were analyzed by using one-way ANOVA followed by Newman–Keuls Multiple comparison test. Data are shown as Mean ± SEM; α, control vs. ISO; β, ISO vs. EE; γ, ISO vs. BF; ^∗∗^*P* < 0.01, ^∗∗∗^*P* < 0.001.

Blood pressure and HR were significantly increased in ISO induced cardiac hypertrophy as compared to normotensive Wistar group (**Figures 2A–C**). The treatment of extract and fraction of *U. wallichiana* significantly decreased the SBP in ISO+EE and ISO+BF compared to diseased control (**Figure 2A**) and it also decreased the HR in ISO+EE and ISO+BF compared to diseased control (**Figure 2D**), respectively. (In support IBP data is given in Supplementary Figures 2A–D.)

### Renin Inhibition

**FIGURE 3:**
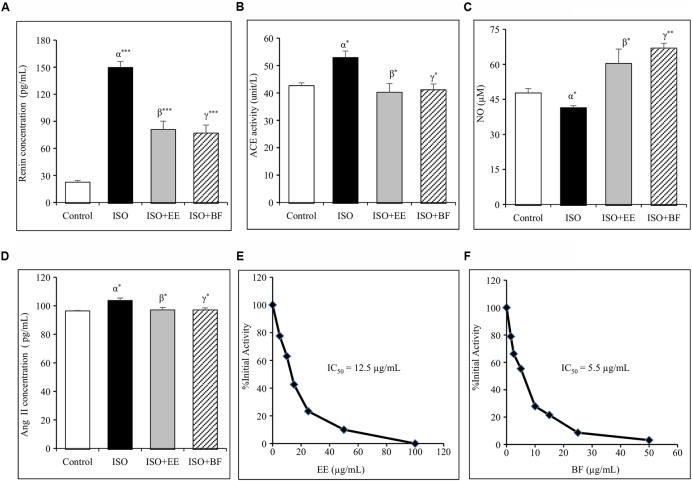
**Effect of *U. wallichiana* on (A)** renin concentration, **(B)** angiotensin converting enzyme (ACE) activity, **(C)** NO concentration, **(D)** Ang II concentration, **(E)** inhibition of human recombinant renin by EE and **(F)** inhibition of human recombinant renin by BF in ISO induced cardiac hypertrophy in wistar rats. Data were analyzed by using one-way ANOVA followed by Newman–Keuls Multiple comparison test. Data are shown as Mean ± SEM; α, control vs. ISO; β, ISO vs. EE; γ, ISO vs. BF; ^∗^*P* < 0.05, ^∗∗^*P* < 0.01, ^∗∗∗^*P* < 0.001.

Renin inhibition assay *in vitro* was conducted to assess the renin inhibitory properties of EE and BF of *U. wallichiana*. The IC_50_ value for EE was found to be 12.5 μg/mL and that of BF was 5.5 μg/mL (**Figures 3E,F**)

### *U. wallichiana* Reduced Plasma Renin Concentration

The plasma renin concentration was increased significantly in ISO group. Administration of EE and BF of *U. wallichiana* significantly decreased plasma renin concentration in ISO+EE and ISO+BF group compared to diseased control (**Figure 3A**) group, respectively.

### *U. wallichiana* Decreased ACE Activity

The plasma ACE activity was increased significantly in ISO group. Further administration of EE and BF of *U. wallichiana* significantly decreased ACE activity in ISO+EE and ISO+BF group compared to diseased control (**Figure 3B**) group, respectively.

### *U. wallichiana* Attenuated AG II Concentration in Plasma

The plasma Ang II level was increased significantly in ISO group. Further administration of EE and BF of *U. wallichiana* significantly decreased ACE activity in ISO+EE and ISO+BF compared to diseased control (**Figure 3D**) group, respectively.

### *U. wallichiana* Increased Plasma Nitrite/Nitrate (NO) Concentration

The concentration of NO was decreased significantly in ISO group. Further administration of EE and BF of *U. wallichiana* significantly increased NO in ISO+EE and ISO+BF group compared to diseased control (**Figure 3C**) group, respectively.

### *U. wallichiana* Increased cGMP Concentration

**FIGURE 4:**
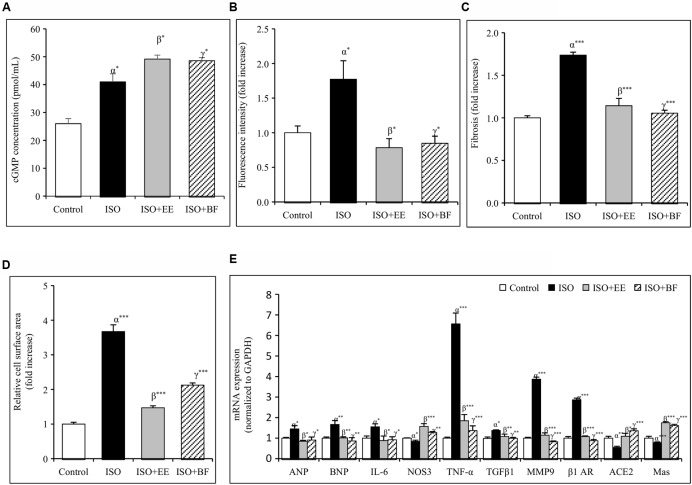
**Effect of *U. wallichiana* on (A)** cGMP concentration, **(B)** fluorescence intensity, **(C)** myocardial fibrosis, **(D)** relative cell surface area, and **(E)** relative mRNA expression level of ANP, BNP, IL-6, NOS3, TNF-α, TGFβ1, MMP9, β1 adrenergic receptor (β1 AR), ACE2 and Mas in ISO induced cardiac hypertrophy in wistar rats. Data were analyzed by using one-way ANOVA followed by Newman–Keuls Multiple comparison test. Data are shown as Mean ± SEM, α, control vs. ISO; β, ISO vs. EE; γ, ISO vs. BF; ^∗^*P* < 0.05, ^∗∗^*P* < 0.01, ^∗∗∗^*P* < 0.001.

The concentration of cGMP was decreased significantly in ISO group. Further administration of EE and BF of *U. wallichiana* significantly increased cGMP concentration in ISO+EE and ISO+BF group compared to diseased control (**Figure 4A**) group, respectively.

### *U. wallichiana* Attenuated ROS Level

The ROS activity was increased significantly in ISO group. Further administration of EE and BF of *U. wallichiana* significantly increased ROS activity in ISO+EE and ISO+BF group compared to diseased control (**Figure 4B**) group, respectively.

### *U. wallichiana* Reduced Expression of Cardiac Hypertrophic Markers

To investigate whether EE and BF of *U. wallichiana* confers cardio-protection in ISO treated group. We have determined the cardiac gene expression of ANP, BNP, TNF-α, IL-6, TGFβ1, MMP9, β1-AR, ACE2, Mas relative to ISO treated group. ISO caused significant up regulation of ANP, BNP, TNF-α, IL-6, TGFβ1, MMP9, β1-AR and down regulation of NOS3, ACE2, Mas as compared to control group. On treatment with the EE and BF of *U. wallichiana* the ANP, BNP, TNF-α, IL-6, TGFβ1, MMP9, β1-AR was significantly reduced in ISO+EE and ISO+BF group and significantly increased in NOS3, ACE2, Mas expression as compared to diseased control group, respectively, (**Figure 4E**).

### *U. wallichiana* Decreased Cardiac Fibrosis and Relative Cell Surface Area

**FIGURE 5:**
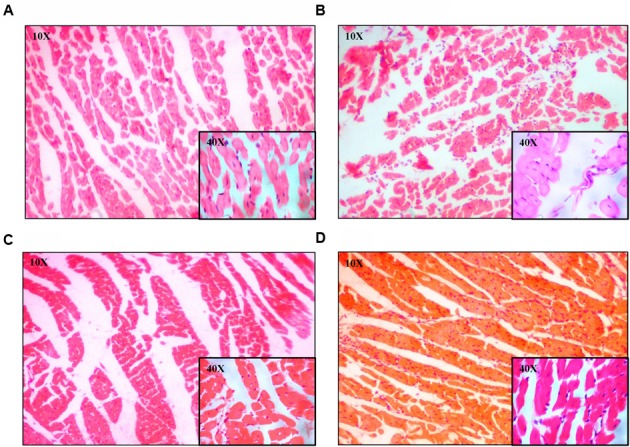
**Representative photomicrographs of heart dyed with haematoxylin and eosin (H&E) stain (10× and 40× magnification). (A)** Control, **(B)** ISO, **(C)** ISO+EE, **(D)** ISO+BF groups, respectively.

The regularly arranged myocardial fibers with clear striations were observed in the control group (**Figure 5A**). The photomicrograph of heart from normal group shows no pathological alteration. The histological sections of the heart obtained from ISO group showed enlargement of cardiomyocyte showing increased cell radius with eccentric positioning of nucleus indicating hypertrophy. Fibrocytic proliferation was also invariably observed along with cellular degenerative changes characterized by scanty cytoplasm and vacuolation. Focal infiltration of inflammatory cells is observed in few animals. There was significant increase in myocardial fibrosis in ISO group (**Figure 5B**). There was also significant increase in relative cell surface area in ISO group. On administration of EE and BF of *U. wallichiana* significantly decreased myocardial fibrosis in ISO+EE and ISO+BF group compared to diseased control (**Figures 4C** and **5C,D**); decreased relative cell surface area in ISO+EE and ISO+BF group as compared to diseased control (**Figures 4D** and **5C,D**) group, respectively. A representative photo has also been given in the Supplementary Figure 4.

## Discussion

In the present study, the *U. wallichiana* attenuated cardiac hypertrophic response induced by ISO, a non-selective β-adrenoceptor agonist. The EE and BF of *U. wallichiana* attenuated cardiac hypertrophy by decreasing SBP and HR and inhibiting plasma renin, ACE, Ang II and augmented plasma NO and cGMP concentration (Supplementary Table 2). The EE and BF of *U. wallichiana* have antihypertensive activity (Syed et al., 2016). ISO in chronic dose not only produced cardiac hypertrophy but also altered the gross structure of the heart. There was an increase in weight of the heart, but no significant effect on weight of the body. The effect of EE and BF of *U. wallichiana* was studied as anti-hypertrophic. The dose of EE (500 mg/kg) and BF (50 mg/kg) of *U. wallichiana* contains quercetin analog flavonoids K058: 9.79 and 1.34 mg, K012: 4.33 and 0.24 mg, K068: 0.625 and 0.15 mg, and K100: 2.49 and 0.41 mg, respectively.

The EE and BF of *U. wallichiana* decreased the heart rate, renin release, and Ang II level there by decreasing the cardiac output. ISO, a directly acting catecholamine, binds with the β-adrenergic receptor in heart and causes significant increase in heart rate (Arnold and McDevitt, 1983), increased plasma renin and Ang II level (Leenen et al., 2001). ISO, a sympathetic β-adrenergic receptor agonist, stimulates renin release via adenylate cyclase-cyclic AMP system (Suzuki and Hashiba, 1986). The stimulatory effect of ISO on renin release was inhibited by the EE and BF of *U. wallichiana* due to the presence of quercetin analog flavonoids. Renin overproduction increases the level of Angiotensin I in the blood, leading to high BP. The IC_50_ value for EE was found to be 12.5 μg/mL and that of BF was 5.5 μg/mL, showing renin inhibition in the present study.

Cardiac mast cell secretes renin initiating the formation of angiotensinogen. Cardiac mast cells releases renin on degranulation by ROS. Renin initiates the activation of a local renin-angiotensin system which culminates in the formation of Ang II (Reid et al., 2011). The quercetin analog flavonoids acts as antioxidant and inhibits release of pro-inflammatory cytokines, IL-6 from mast cell. Therefore the EE and BF of *U. wallichiana* attenuates the ROS production, due to its antioxidant activity, inhibiting the TNF-α and IL-6 and it has renin inhibition property (**Figures 3E,F**). Thus EE and BF may be responsible to inhibit the release of renin from the cardiac mast cells.

Angiotensin converting enzyme, an enzyme that regulates RAAS pathway, contains zinc and a zinc ion (Ojeda et al., 2010). The flavonoids quercetin analog forms chelate complex with zinc and inhibit the ACE enzyme (Actis-Goretta et al., 2006; Guerrero et al., 2012). The ACE2 and Mas receptors are involved in the cardiac hypertrophy as their reduced expression is found in hypertrophied heart (Zhang et al., 2014). ACE2, by degradation of Ang II, forms Ang (1–7) which opposes actions of Ang II by binding to the Mas receptor leading to vasodilation, anti-hypertrophic and anti-fibrotic effect (Dias-Peixoto et al., 2012). The EE and BF of *U. wallichiana* increased the ACE2 and Mas receptor gene expression as compared to the ISO group.

In RAS pathway, increased renin and ACE enzyme causes increased level of Ang II. Angiotensin II activates NAD(P)H oxidase leading to oxidative stress (Veresh et al., 2008) causing decrease in bioavailability of NO resulting in endothelial dysfunction and vasoconstriction of arterioles leading to hypertension. Emerging evidence suggests that the nitricoxide (NO)/guanosine 3′5′-cyclic monophosphate (cGMP) pathway plays an important role in the modulation of cardiac hypertrophy (Ozaki et al., 2002). Therefore the EE and BF of *U. wallichiana* increased the bioavailability of NO/cGMP leading to vasodilation and act as anti-hypertrophic (Tirziu and Simons, 2008). As NO has anti-hypertrophic effect which is mediated by cGMP which involves cGMP-dependent protein kinase I activation. Plasma NO level was decreased by ISO, an indirect indicator of endogenous NO production.

NO/sGC/cGMP/cGMP dependent protein kinase G acts as cardioprotective. *U. wallichiana* causes increase in the NO level, probably via signal transduction pathway which results in cGMP generation. Cardiovascular diseases are associated with the impairment of Nitric oxide/soluble guanylate cyclase/cGMP signaling (Korkmaz et al., 2009). NO/cGMP/ANP act as anti-hypertrophic. ANP and BNP are present in cardiac tissues (London, 2006), and act as cardiac hypertrophic markers (Althurwi et al., 2013).

The cardiac mast cell induces MMP activation due to oxidative stress as well as TNF-α which causes degradation of collagen causing cardiac fibrosis in cardiac hypertrophy (Lee et al., 2010; Parthasarathy et al., 2014). ROS also have potent effects on the extracellular matrix, stimulating cardiac fibroblast proliferation and activating MMPs. EE and BF of *U. wallichiana* attenuates the increased expression of TGFβ1, MMP9, and ROS activity. TNF-α, IL-6 are cytokines which mediates various physiological and pathological functions like inflammation, cellular survival, growth, differentiation, and apoptosis. TNF-α in the myocardium produce inflammation and apoptosis via TNFR1 receptor (Sack et al., 2000) where as IL-6 also produce cardiac hypertrophy, fibrosis, and inflammation (Diaz et al., 2009; Meléndez et al., 2010). The flavonoids and quercetin analogs present in EE and BF of *U. wallichiana* act as antioxidant (LaManna et al., 2011; Brunetti et al., 2013) thus decreasing ROS activity.

## Conclusion

**FIGURE 6:**
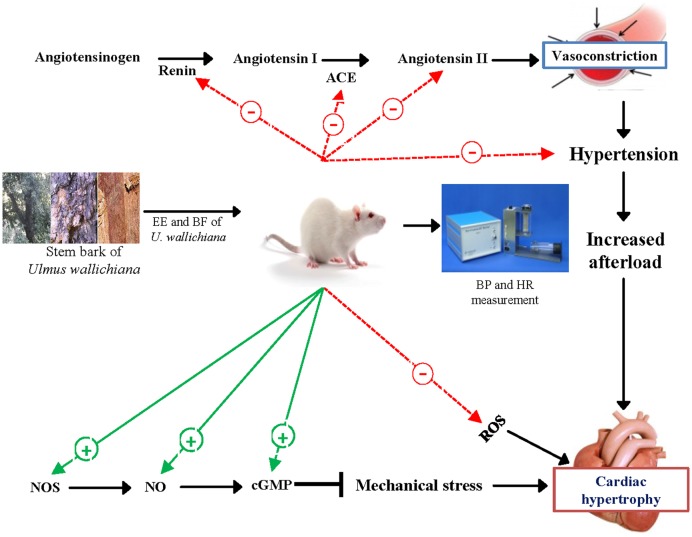
**Schematic diagram showing the mechanism of action of EE and BF of *U. wallichiana* in ISO induced cardiac hypertrophy.** ACE, angiotensin converting enzyme; cGMP, cyclic guanosine monophosphate; NOS, nitric oxide synthase.

We suggest that the quercetin analog flavonoids present in the EE and BF of *U. wallichiana* attenuated cardiac hypertrophy (**Figure 6**) showing cardioprotective effect. The BF showed pharmacological response and potent at a lower dose (50 mg/kg/day) as compared to EE (500 mg/kg/day) as BF contains higher concentration of flavonoids than EE (Sharan et al., 2010).

Thus, the EE and BF of *U. wallichiana* showed cardioprotective effect against ISO induced cardiac hypertrophy. Based on these findings the marker compounds present in *U. wallichiana* can be isolated to study the mechanism of action and it may be a novel therapeutic agent for treatment of cardiac hypertrophy.

## Author Contributions

AS, KH, and JG designed the research work; AS, SL, DM, GV, AG, and SK performed the research experiments; RM, HB, KH, and JG contributed new reagents/analytic tools; AS, SL, DM, KH, HB, RM, and JG analyzed data; AS, KH, and JG wrote the paper.

## Conflict of Interest Statement

The authors declare that the research was conducted in the absence of any commercial or financial relationships that could be construed as a potential conflict of interest.

## Abbreviations

BF: butanolic fraction
BNP: brain natriuretic peptide
BP: blood pressure
cGMP: cyclic guanosine monophosphate
DBP: diastolic blood pressure
DCFDA: 2′,7′-Dichlorodihydrofluorescein diacetate
DCF: 2′,7′-Dichlorofluorescein
EE: ethanolic extract
FAP: N-[3-(2-furyl) acryloyl]-L-phenylalanine
GG: glycylglycine
HF: heart failure
HR: heart rate
IBP: invasive blood pressure
IL-6: Interleukin 6
ISO: isoprenaline
MAP: mean arterial pressure
NIBP: non-invasive blood pressure
NO: total nitrate/nitrite
qPCR: quantitative polymerase chain reaction
RAAS: Renin-angiotensin-aldosterone system
ROS: reactive oxygen species
SBP: systolic blood pressure
TNF-α: tumor necrosis factor alpha
