# Supplementary material for: Cardioprotective Effect of Ulmus wallichiana Planchon in β-Adrenergic Agonist Induced Cardiac Hypertrophy
Source: Front Pharmacol. 2016 Dec 21;7:510. doi: 10.3389/fphar.2016.00510 (PMC5174112; doi:10.3389/fphar.2016.00510)
Supplement: Supplementary file 1 [file Presentation_1.pptx]

## Slide 1
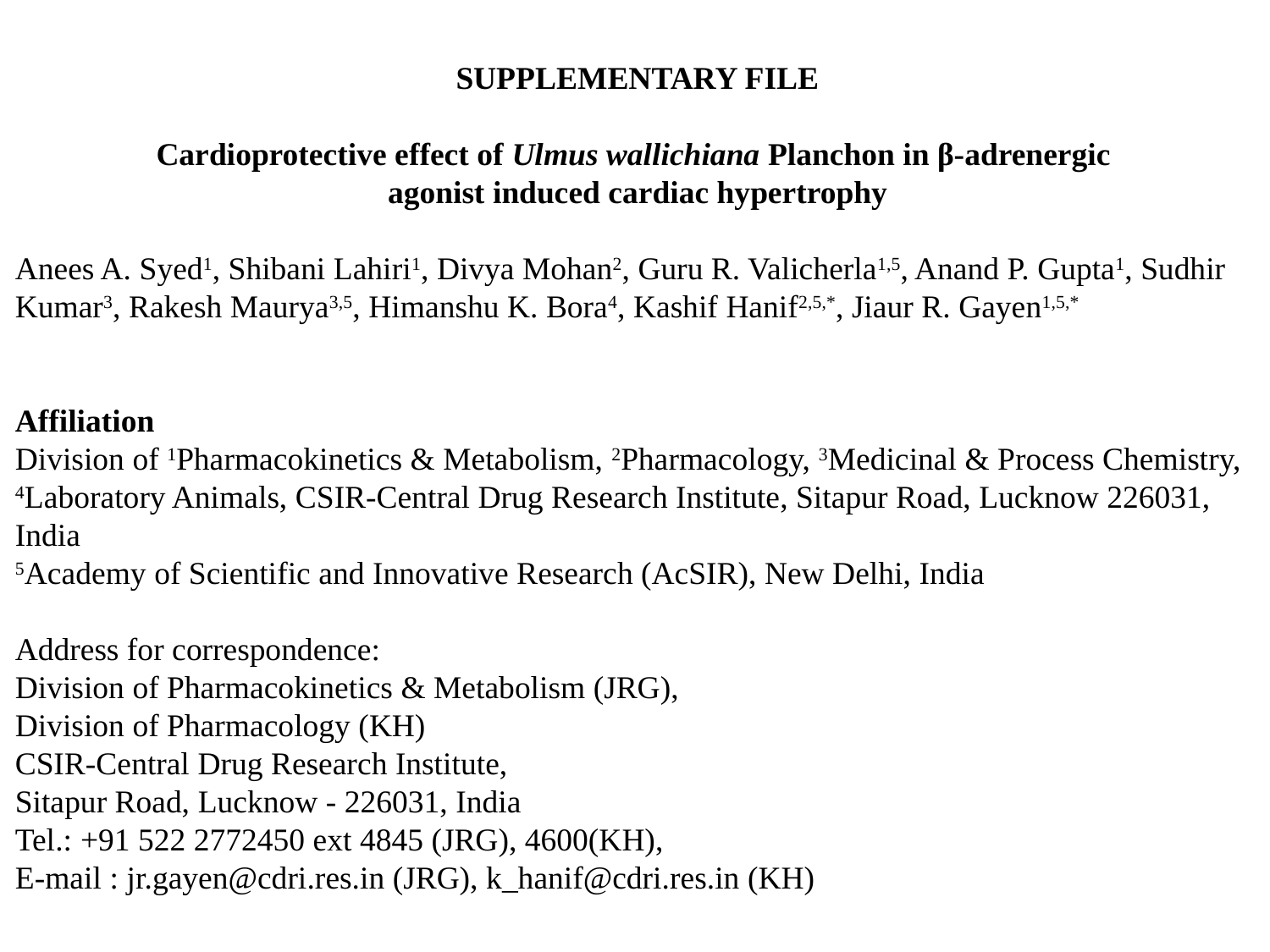

SUPPLEMENTARY FILE
Cardioprotective effect of Ulmus wallichiana Planchon in β-adrenergic
agonist induced cardiac hypertrophy
Anees A. Syed1, Shibani Lahiri1, Divya Mohan2, Guru R. Valicherla1,5, Anand P. Gupta1, Sudhir Kumar3, Rakesh Maurya3,5, Himanshu K. Bora4, Kashif Hanif2,5,*, Jiaur R. Gayen1,5,*
Affiliation
Division of 1Pharmacokinetics & Metabolism, 2Pharmacology, 3Medicinal & Process Chemistry, 4Laboratory Animals, CSIR-Central Drug Research Institute, Sitapur Road, Lucknow 226031, India
5Academy of Scientific and Innovative Research (AcSIR), New Delhi, India
Address for correspondence:
Division of Pharmacokinetics & Metabolism (JRG),
Division of Pharmacology (KH)
CSIR-Central Drug Research Institute,
Sitapur Road, Lucknow ‐ 226031, India
Tel.: +91 522 2772450 ext 4845 (JRG), 4600(KH),
E-mail : jr.gayen@cdri.res.in (JRG), k_hanif@cdri.res.in (KH)

## Slide 2
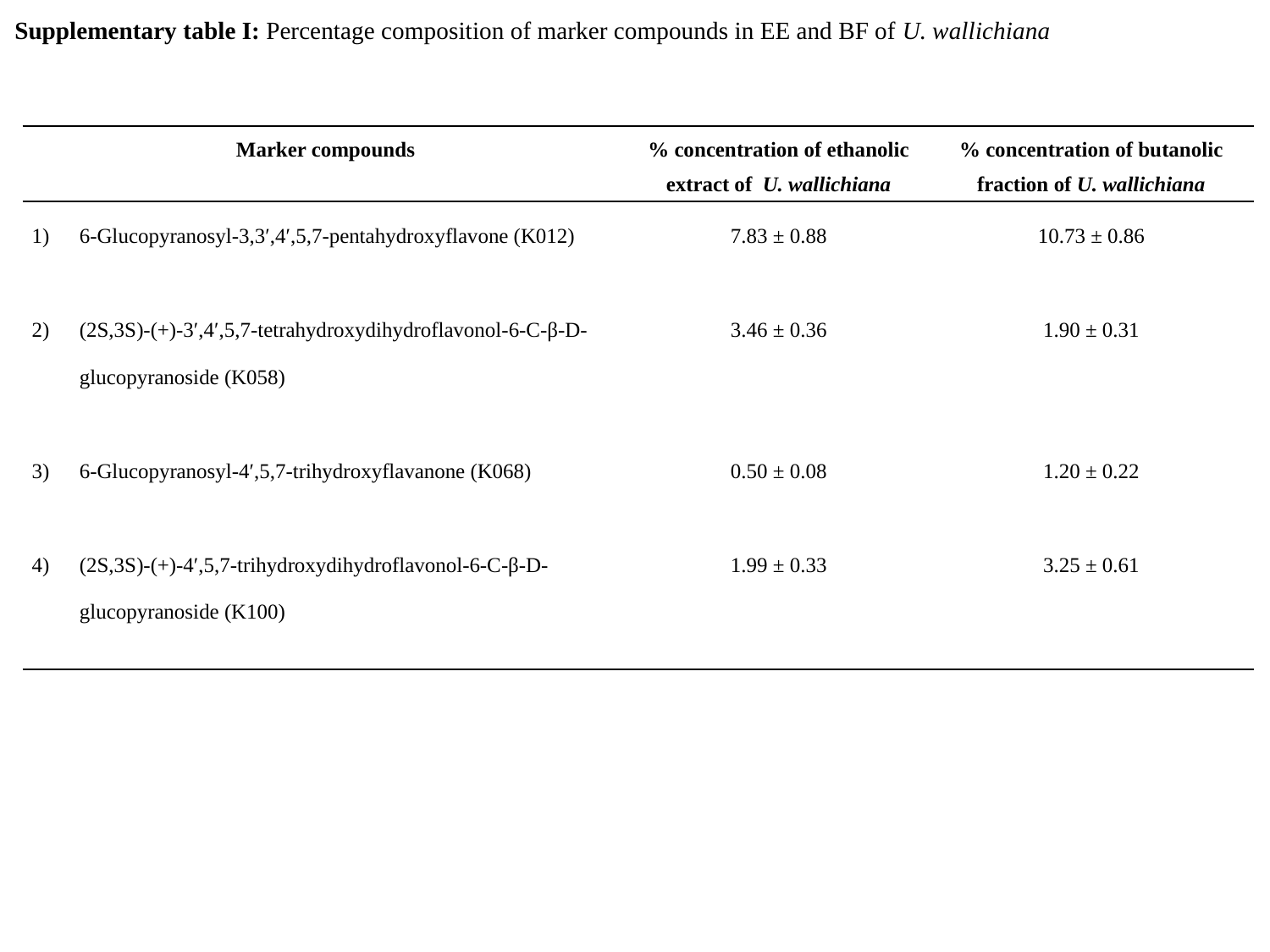

Supplementary table I: Percentage composition of marker compounds in EE and BF of U. wallichiana
| Marker compounds | % concentration of ethanolic extract of U. wallichiana | % concentration of butanolic fraction of U. wallichiana |
| --- | --- | --- |
| 6-Glucopyranosyl-3,3′,4′,5,7-pentahydroxyflavone (K012) (2S,3S)-(+)-3′,4′,5,7-tetrahydroxydihydroflavonol-6-C-β-D-glucopyranoside (K058) 6-Glucopyranosyl-4′,5,7-trihydroxyflavanone (K068) (2S,3S)-(+)-4′,5,7-trihydroxydihydroflavonol-6-C-β-D-glucopyranoside (K100) | 7.83 ± 0.88   3.46 ± 0.36     0.50 ± 0.08   1.99 ± 0.33 | 10.73 ± 0.86   1.90 ± 0.31     1.20 ± 0.22   3.25 ± 0.61 |

## Slide 3
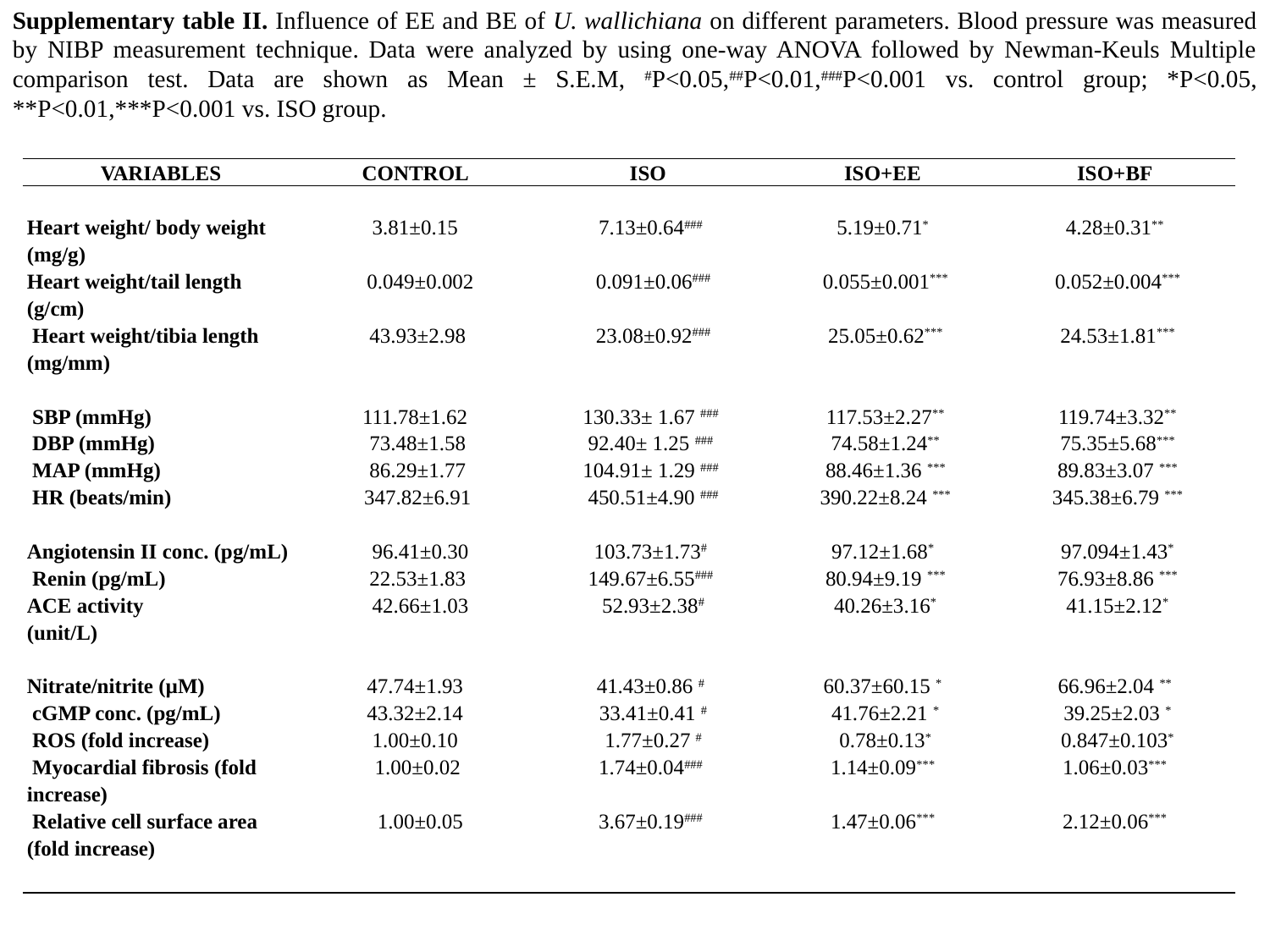

Supplementary table II. Influence of EE and BE of U. wallichiana on different parameters. Blood pressure was measured by NIBP measurement technique. Data were analyzed by using one-way ANOVA followed by Newman-Keuls Multiple comparison test. Data are shown as Mean ± S.E.M, #P<0.05,##P<0.01,###P<0.001 vs. control group; *P<0.05, **P<0.01,***P<0.001 vs. ISO group.
| VARIABLES | CONTROL | ISO | ISO+EE | ISO+BF |
| --- | --- | --- | --- | --- |
| Heart weight/ body weight (mg/g) Heart weight/tail length (g/cm)  Heart weight/tibia length (mg/mm)  SBP (mmHg)  DBP (mmHg)  MAP (mmHg)  HR (beats/min) Angiotensin II conc. (pg/mL)  Renin (pg/mL) ACE activity (unit/L) Nitrate/nitrite (µM)  cGMP conc. (pg/mL)  ROS (fold increase)  Myocardial fibrosis (fold increase)  Relative cell surface area (fold increase) | 3.81±0.15    0.049±0.002    43.93±2.98     111.78±1.62  73.48±1.58  86.29±1.77  347.82±6.91    96.41±0.30  22.53±1.83  42.66±1.03     47.74±1.93 43.32±2.14 1.00±0.10 1.00±0.02  1.00±0.05 | 7.13±0.64###    0.091±0.06###    23.08±0.92###     130.33± 1.67 ### 92.40± 1.25 ### 104.91± 1.29 ###  450.51±4.90 ###   103.73±1.73# 149.67±6.55###  52.93±2.38#     41.43±0.86 #  33.41±0.41 #  1.77±0.27 # 1.74±0.04### 3.67±0.19### | 5.19±0.71\*    0.055±0.001\*\*\*    25.05±0.62\*\*\*      117.53±2.27\*\*  74.58±1.24\*\*  88.46±1.36 \*\*\*  390.22±8.24 \*\*\*   97.12±1.68\*  80.94±9.19 \*\*\*  40.26±3.16\*     60.37±60.15 \*  41.76±2.21 \*  0.78±0.13\* 1.14±0.09\*\*\* 1.47±0.06\*\*\* | 4.28±0.31\*\*    0.052±0.004\*\*\*    24.53±1.81\*\*\*      119.74±3.32\*\*  75.35±5.68\*\*\*  89.83±3.07 \*\*\*  345.38±6.79 \*\*\*    97.094±1.43\*  76.93±8.86 \*\*\*  41.15±2.12\*   66.96±2.04 \*\*  39.25±2.03 \*  0.847±0.103\* 1.06±0.03\*\*\* 2.12±0.06\*\*\* |

## Slide 4
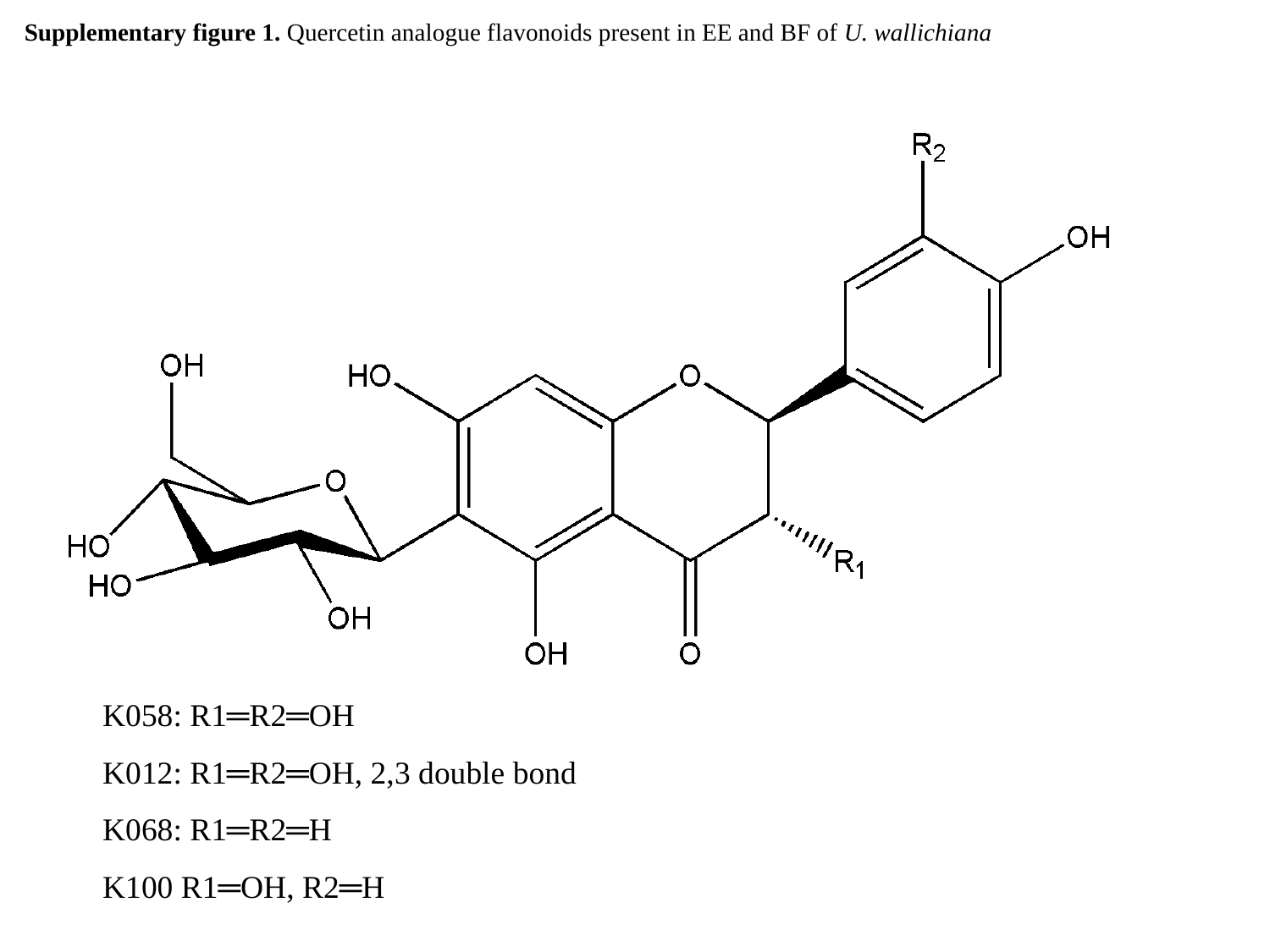

Supplementary figure 1. Quercetin analogue flavonoids present in EE and BF of U. wallichiana
K058: R1═R2═OH
K012: R1═R2═OH, 2,3 double bond
K068: R1═R2═H
K100 R1═OH, R2═H

## Slide 5
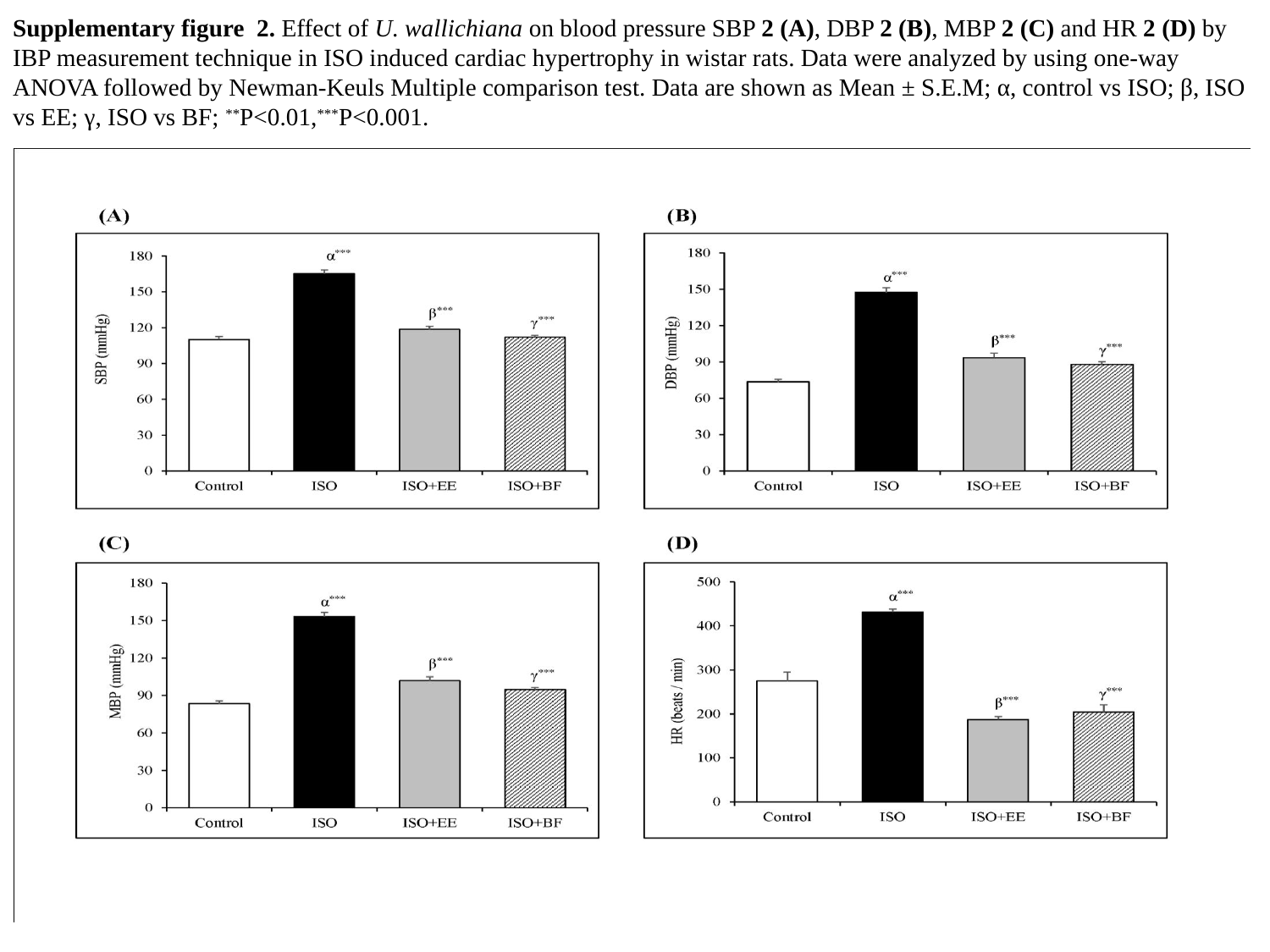

Supplementary figure 2. Effect of U. wallichiana on blood pressure SBP 2 (A), DBP 2 (B), MBP 2 (C) and HR 2 (D) by IBP measurement technique in ISO induced cardiac hypertrophy in wistar rats. Data were analyzed by using one-way ANOVA followed by Newman-Keuls Multiple comparison test. Data are shown as Mean ± S.E.M; α, control vs ISO; β, ISO vs EE; γ, ISO vs BF; **P<0.01,***P<0.001.

## Slide 6
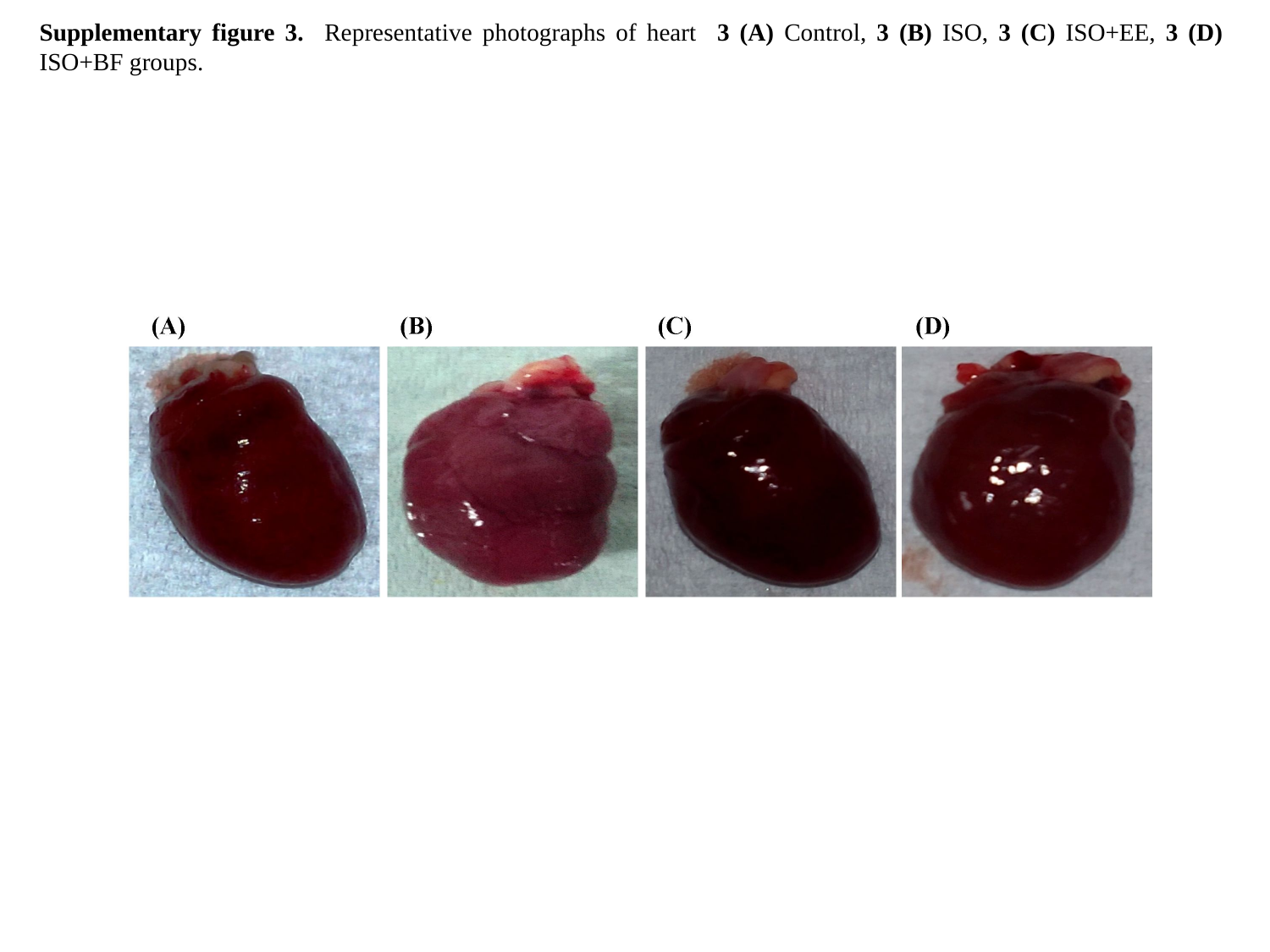

Supplementary figure 3. Representative photographs of heart 3 (A) Control, 3 (B) ISO, 3 (C) ISO+EE, 3 (D) ISO+BF groups.

## Slide 7
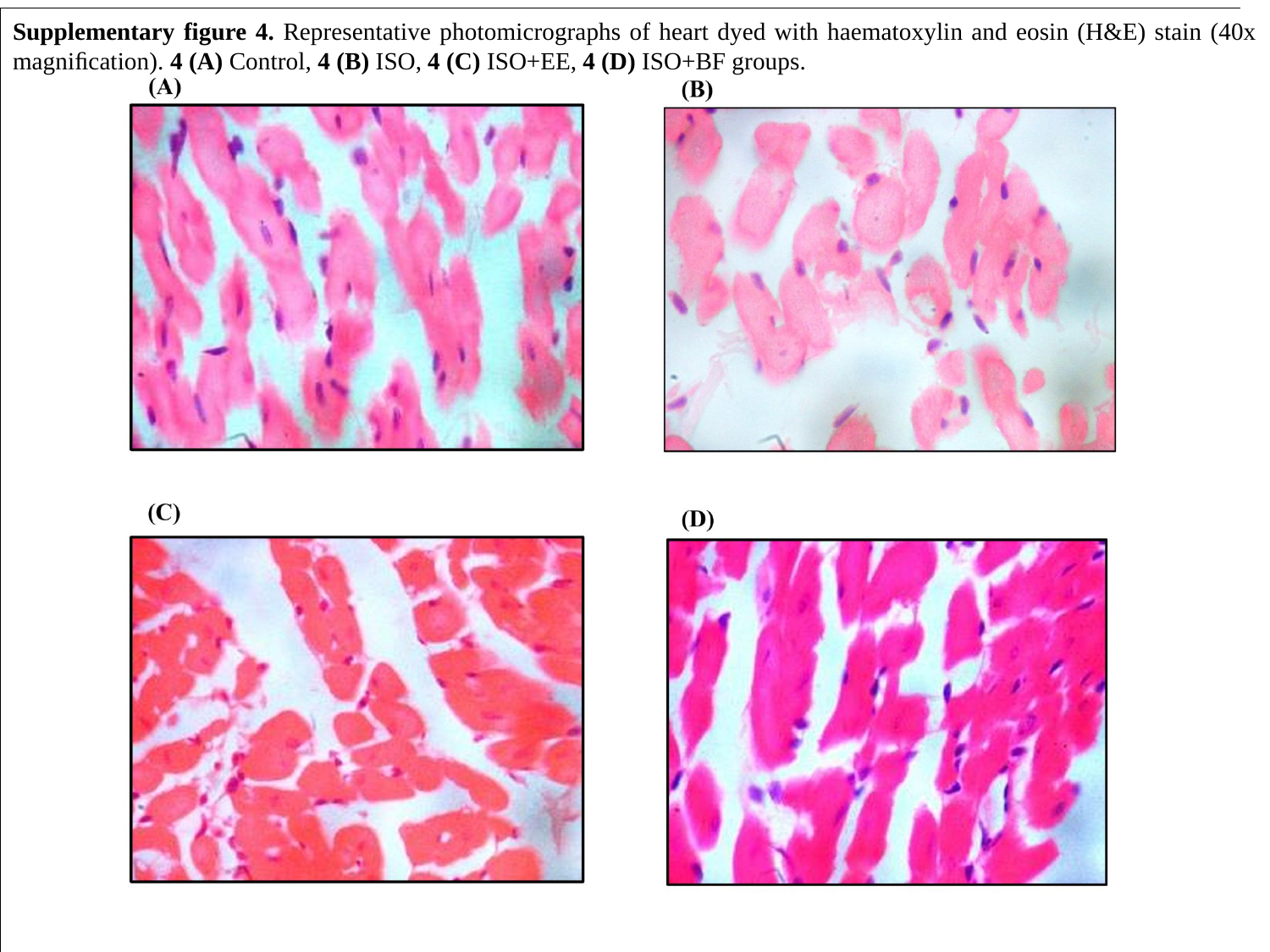

Supplementary figure 4. Representative photomicrographs of heart dyed with haematoxylin and eosin (H&E) stain (40x magniﬁcation). 4 (A) Control, 4 (B) ISO, 4 (C) ISO+EE, 4 (D) ISO+BF groups.
